# Antibiotic Susceptibility Profile of *Aeromonas* Species Isolated from Wastewater Treatment Plant

**DOI:** 10.1100/2012/764563

**Published:** 2012-08-13

**Authors:** Isoken H. Igbinosa, Anthony I. Okoh

**Affiliations:** Applied and Environmental Microbiology Research Group (AEMREG), Department of Biochemistry and Microbiology, University of Fort Hare, Private Bag X1314, Alice 5700, South Africa

## Abstract

This study assessed the prevalence of antibiotic-resistant *Aeromonas* species isolated from Alice and Fort Beaufort wastewater treatment plant in the Eastern Cape Province of South Africa. Antibiotic susceptibility was determined using the disc diffusion method, and polymerase chain reaction (PCR) assay was employed for the detection of antibiotics resistance genes. Variable susceptibilities were observed against ciprofloxacin, chloramphenicol, nalidixic acid, gentamicin, minocycline, among others. *Aeromonas* isolates from both locations were 100% resistant to penicillin, oxacillin, ampicillin, and vancomycin. Higher phenotypic resistance was observed in isolates from Fort Beaufort compared to isolates from Alice. Class A *pse*1 **β**-lactamase was detected in 20.8% of the isolates with a lower detection rate of 8.3% for *bla_TEM_* gene. Class 1 integron was present in 20.8% of *Aeromonas* isolates while class 2 integron and *TetC* gene were not detected in any isolate. The antibiotic resistance phenotypes observed in the isolates and the presence of **β**-lactamases genes detected in some isolates are of clinical and public health concern as this has consequences for antimicrobial chemotherapy of infections associated with *Aeromonas* species. This study further supports wastewater as potential reservoirs of antibiotic resistance determinants in the environment.

## 1. Introduction

Wastewater environment is considered a significant reservoir of antibiotic-resistant bacteria [1, 2]. Nutrient-rich environments like sewage and wastewater create optimal conditions to promote horizontal gene transfer processes [3, 4]; hence wastewater environment is regarded as hotspot for spread of antibiotic resistance determinant [1, 3, 5–7]. The significance of municipal wastewater treatment plants as sources of antimicrobial resistance determinants and the risks of contamination of surface waters have been documented in numerous studies [8–10]. Wastewater could be a source of surface and ground water contamination which may result in spread of antimicrobial resistance determinants to drinking and consequently to consumers. Studies have documented the detection of antimicrobial resistance in wastewater and drinking water [11–13]. Therefore, ubiquitous bacteria, which are capable of colonizing different water types, are of particular interest to assessing potential forms of antimicrobial resistance dissemination. Given their ubiquity in water environment and patterns of acquired antimicrobial resistance, members of the genus *Aeromonas* are good examples of such bacteria. Regardless of the ubiquity of aeromonads in aquatic environments and the possibility to develop antimicrobial resistance, the patterns of resistance of *Aeromonas* genus present in wastewater are not fully documented in scientific literature. 

As early as antibiotics have been in use, microbial antibiotic resistance was developed. *β*-lactam antibiotics are commonly used in the treatment of bacterial infections but they are hydrolysed by *β*-lactamase enzymes produced by resistant bacteria [14, 15]. Presently, hundreds of *β*-lactamase have been identified and classified based on molecular or functional characteristics [15–17]. Also, class I integrons are usually reported to contain antibiotic-resistant gene cassettes and related with other mobile elements such as plasmids, which could contribute to the dissemination of resistance genes [18]. The purpose of this study was to assess the antibiotic resistance profiles of *Aeromonas* species isolated from wastewater in the Eastern Cape Province of South Africa as part of our surveillance of antibiotic resistance reservoir in the environment; our major objective was to (i) isolate and identify *Aeromonas* species, (ii) elucidate antibiotic characteristics of the isolates, and (iii) to screen the *Aeromonas* isolates for associated integron and antibiotic resistance genes.

## 2. Material and Methods

### 2.1. Isolation of *Aeromonas*

To isolate *Aeromonas* species, wastewater was collected from mixed liquor compartment of Fort Beaufort (geographical coordinates: S 32° 47.071′ E026° 38.916′) and Alice (geographical coordinates: S 32° 46.629′ E026° 50.149′) wastewater treatment plants in the Eastern Cape Province of South Africa. Samples were collected into sterile 1 L sampling bottles and placed in a cooler box and transported to the laboratory for analyses. On arrival in the laboratory, 100 *μ*L of the undiluted and diluted samples were spread on several Glutamate Starch Phenol-red (GSP) agar plates and incubated at 37°C for 24 h. Typical yellow colonies were randomly selected as presumptive *Aeromonas* isolates, purified, and transferred to nutrient agar plate. The pure isolates were subjected to Gram staining and oxidase and catalase test. Only Gram-negative, oxidase and catalase positive isolates were selected for biochemical identification using API 20 NE kit. The strips were then read, and final identification was made using API lab plus software (bioMerieux, Marcy l'Etoile, France). 

### 2.2. Detection of Antibiotic Resistance Phenotypes

The susceptibilities of the identified *Aeromonas *species to 20 antibiotics were determined using disc diffusion method as described elsewhere [19, 20]. The antibiotics used include Ciprofloxacin (5 *μ*g), Trimethoprim (5 *μ*g), Chloramphenicol (3 *μ*g), Penicillins (10 *μ*g), Clindamycins (2 *μ*g), Ofloxacin (*μ*g), Ampicillin-sulbactam (20 *μ*g), Oxacillin (1 *μ*g). Ampicillin (25 *μ*g), Gentamicin (10 *μ*g), Nalidixic acid (30 *μ*g), Cefotaxime (30 *μ*g), Nitrofurantoin (300 *μ*g), Sulphamethoxazole (25 *μ*g), Cephalothin (30 *μ*g), Erythromycin (15 *μ*g), Tetracycline (10 *μ*g), Minocycline (30 *μ*g), Vancomycin (30 *μ*g), Rifamycin (5 *μ*g), antibiotic disks were purchased from Mast Diagnostics (Mast Group Merseyside UK). Isolates were identified as susceptible, intermediate or, resistant according to the CLSI (2006) guidelines.

### 2.3. Isolation of Genomic DNA

DNA was extracted following the method of Sambrook and Russell [21]. Briefly, single colonies of the bacteria strains grown overnight at 37°C on nutrient agar plates were picked, suspended in 500 mL of sterile Milli-Q PCR grade water (Merck, SA), and the cells were lysed using Dri-block DB.2A (Techne, SA) for 10 min at 100°C. The cell debris was removed by centrifugation at 11,000 g for 5 min using a minispin microcentrifuge (Merck, SA) and immediately placed on ice; the supernatant was used directly as template DNA or stored at −20°C until ready for use.

### 2.4. PCR Detection of Antibiotic Resistance Genes

Polymerase chain reaction (PCR) was used to detect antibiotic-resistant genes in the *Aeromonas* species using the specific primer pairs for *pse1*, *bla*_*TEM*_, *TetC*, class 1 integron, class 2 integron as shown in Table 1. All reactions were carried out in 25 *μ*L volume of reaction buffer containing 0.05 unit/*μ*L Taq polymerase as directed by the manufacturer (Fermentas Life Sciences). Cycling conditions (Bio-Rad My Cycler Thermal Cycler) were as follows; *pse1-PSE-1/CARB-2* (*blaP1* class A *β*-lactamase) (initial denaturation at 96°C for 5 min, then 30 cycles of denaturation at 96°C for 30 s, annealing at 60°C and a single extension of 5 min at 72°C); class 1 and class 2 integron (initial denaturation at 94°C for 2 min followed by denaturation at (95°C for 45 s), annealing (56°C for 1 min), and extension (72°C for 90 s) for 30 cycles and a final amplification cycle at 72°C for 10 min); *bla*_*TEM*_ (3 min at 93°C, 40 cycles of 1 min at 93°C, 1 min at 55°C and 1 min at 72°C and finally 7 min at 72°C), *TetC* (3 min at 94°C, followed by 30 cycles of 1 min at 94°C, 1 min at 65°C and 1 min at 72°C followed by 10 min at 72°C). Electrophoresis of amplicons was performed with 1% agarose gel (Hispanagar, Spain) containing Ethidium Bromide (EtBr) (Merck, SA) with 0.5 mg/L for 1 h at 100 V in 0.5 × TAE buffer (40 mM Tris- HCl, 20 mM Na-acetate, 1 mM EDTA, pH 8.5) and visualized under an UV transilluminator system Alliance 4.7 XD-79 (UVITEC Cambridge).

## 3. Results

Twenty-four *Aeromonas* isolates (18 from Fort Beaufort WWTP and 6 from Alice WWTP) were identified using API 20 NE. All the isolates were resistant to ampicillin, oxacillin, and vancomycin. Higher percentages of isolates from Alice showed susceptibilities to the antibiotics than isolates from Fort Beaufort except against clindamycin which had 33.3% susceptibility from isolates in Fort Beaufort and 16.7% susceptibility from isolates in Alice. *Aeromonas* isolates from Fort Beaufort showed highest susceptibilities against chloramphenicol (61.1%), gentamicin, nitrofurantoin and cefotaxime (55.6%), and minocycline (50%), while high resistances were demonstrated against cephalothin (94.4%) and tetracycline (77.8%) as shown in Figure 3. On the other hand, isolates from Alice showed 83.3% susceptibility against ciprofloxacin, chloramphenicol, ofloxacin, gentamicin, and nalidixic acid and 66.7% susceptibility against nitrofurantoin and erythromycin as shown in Figure 4.

### 3.1. Detection of Antibiotic Resistant Gene

**Figure 1:**
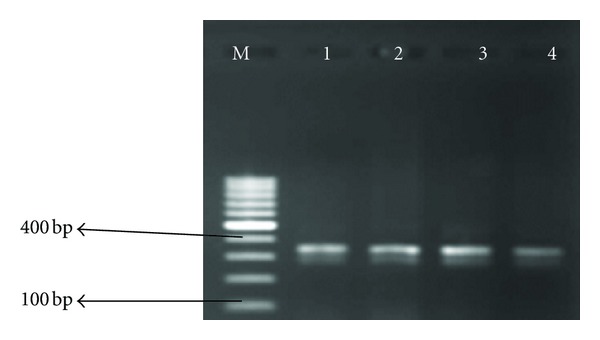
Agarose gel electrophoresis of amplicons of positive *Aeromonas* isolates for pse1-PSE-1/CARB-2 (blaP1 class A *β*-lactamase) Lane M = DNA ladder 100 bp, Lanes 1–4 *Aeromonas* isolates. Expected amplicon size 321 bp.

**Figure 2:**
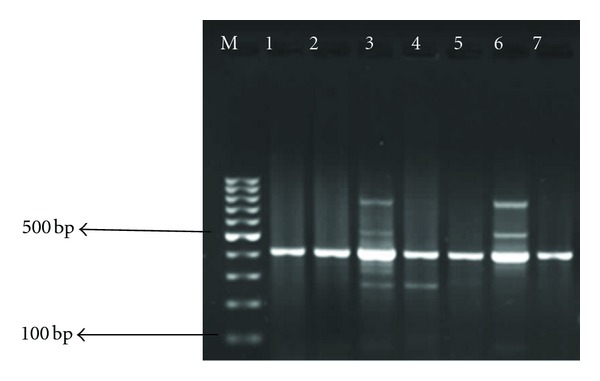
Agarose gel electrophoresis of PCR products of encoded class 1 integron from positive *Aeromonas* strains. Lane M = DNA ladder, Lanes 1–7 *Aeromonas * isolates.

**Figure 3:**
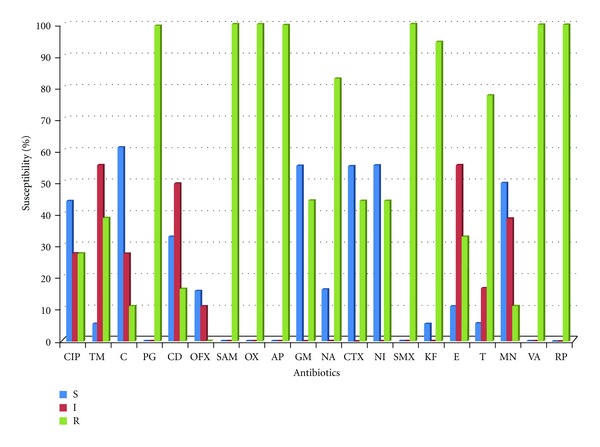
Antibiotic susceptibility of isolates from Fort Beaufort wastewater treatment plant. CIP-Ciprofloxacin, TM-Trimethoprim, C-Chloramphenicol,PG-penicillin, CD-Clindamycin, OFX-Ofloxacin, SAM-Ampicillin-sulbactam, OX-Oxacillin, AP-Ampicillin, GM-Gentamicin, NA-Nalidixic acid, CTX-Cefotaxime, NI-Nitrofurantoin, SMX-Sulphamethoxazole, KF-Cephalothin, E-Erythromycin,T-Tetracycline, MN- Minocycline, VA-Vacomycin, RP-Rifamycin.

**Figure 4:**
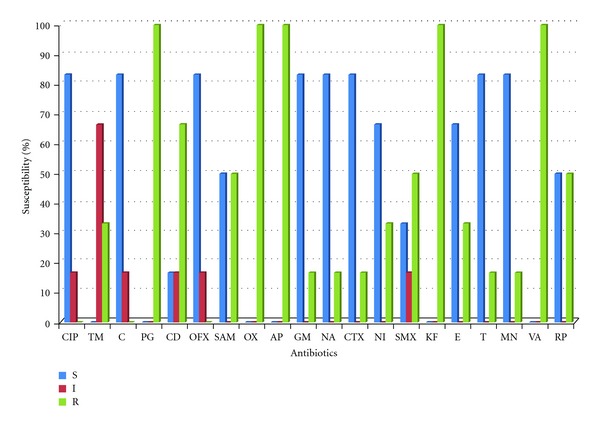
Antibiotic susceptibility of isolates from Alice wastewater treatment plant. CIP-Ciprofloxacin, TM-Trimethoprim, C-Chloramphenicol,PG-penicillin, CD-Clindamycin, OFX-Ofloxacin, SAM-Ampicillin-sulbactam, OX-Oxacillin, AP-Ampicillin, GM-Gentamicin, NA-Nalidixic acid, CTX-Cefotaxime, NI-Nitrofurantoin, SMX-Sulphamethoxazole, KF-Cephalothin, E-Erythromycin,T-Tetracycline, MN- Minocycline, VA-Vacomycin, RP-Rifamycin, S-susceptibility, I- intermediate, R-resistance.

In general class 1 integron was detected in 20.8% of *Aeromonas* isolates from wastewater samples, while class 2 integron was not detected in any isolate. Furthermore, class A *β*-lactamase gene was detected in 20.8% of isolates while *bla*_*TEM*_ gene was present in 8.3% of *Aeromonas* isolates, but *TetC* was not detected in any of the *Aeromonas* isolates. Figures 1 and 2 show gel electrophoresis of PCR products of *pse1-PSE-1/CARB-2* (*blaP1* class A *β*-lactamase) and class 1 integron, respectively, while Table 2 shows the distribution of antibiotic resistance genes.

## 4. Discussion

Antibiotic resistance of *Aeromonas* species to multiple antibiotics is becoming a serious public health concern as revealed in this study. Absolute resistance of *Aeromonas* to ampicillin and oxacillin was observed in this study which may be attributed to *β*-lactamase activity in the resistant isolates. Resistance was observed against tetracycline especially amongst isolates from Fort Beaufort samples. Tetracycline resistance has been reported in *Aeromonas* species isolated from a river that receives wastewater discharge [25]. Similarly, *Aeromonas hydrophila*, *Aeromonas caviae*, and *Aeromonas veronii* isolated from human diarrhoeic stool in Mexico [26] showed variable resistances to tetracycline. Jacobs and Chenia [27] also observed high resistance to tetracycline in *Aeromonas* species from aquaculture system in South Africa. Furthermore, variable resistance of *Aeromonas* isolates to other antibiotics was observed in the study which includes erythromycin, nalidixic acid, gentamicin, among others. Comparable antibiotic resistance pattern in *Aeromonas* has been documented in South Africa. Obi et al. [28] documented similar resistance profile in *Aeromonas* species isolated from diarrhoeic stools of patients in Vhembe district, Limpopo, South Africa.

Integrons are genetic elements that enable bacteria to acquire and express gene cassettes; most of them are involved in antibiotic resistance [29]. Class 1 integron was present in some of the isolates in this study, while class 2 integron was detected in these isolates. Similar findings of the presence of class 1 integron have been documented; for example,Jacobs and Chenia [27] reported the presence of class 1 integron in *Aeromonas *species isolated from South African aquaculture system, and in that study class 2 integron was not detected. The detection of class 1 integron may be attributed to a wide spread distribution of class 1 integron in Gram-negative microorganism. Pérez-Valdespino et al. [26] reported presence of class 1 integron in *Aeromonas* species isolated from patient with diarrhoea in Mexico and found it had association with the acquisition of resistance to antimicrobials in *Aeromonas* species. The presence of class1 and 2 integron in *Aeromonas* and Enterobacteriaceae in Portugal was also reported [30, 31]; however higher prevalence of class 1 integron was found in final effluent [30, 31]. The presence of class 1 integron in final effluent is alarming potentiating wastewater treatment plant as reservoir for horizontal gene transfer for the selection of antimicrobial resistance genes among aquatic organisms in the environment. Consequently, wastewater treatment plant effluent discharges into the receiving waterbodies pose a threat to the environment. 

Tetracycline resistance gene (*TetC*) was not detected in this study in corroboration of the report of Jacobs and Chenia[27], on *Aeromonas* species isolated from South African aquaculture system. The absence of *TetC* gene may not exclude the presence of other *tet* resistance gene determinants; however, only *TetC* determinant was assayed for in the present study. Structurally, *β*-lactamases are classified into four, which are class A, B, C, and D. Class A *β*-lactamase *pse1 *gene was detected in low concentration in these *Aeromonas* isolates. The detection of *pse1*gene in class 1 integron gene cassettes of *Aeromonas* species isolated from South African aquaculture systems has been documented [27]. The presence of*pse1-PSE-1/CARB-2 (blaP1 *class A *β*-lactamase) in wastewater treatment plant mixed liquor and aquaculture systems in South Africa may suggest a wide distribution of *pse1 *gene in South African aquatic ecosystem, though further research is needed to validate this hypothesis. *β*-lactam antibiotics is one of the choice antibiotics for the treatment of bacterial infections; however their efficiency has greatly deteriorated due to the production of *β*-lactamases by resistant bacterial strains. The presence of *β*-lactamase gene in *Aeromonas* has been reported in several studies. Recently, some studies have reported the detection of *bla*_*TEM*_-1 gene in Gram-negative isolates resistant to ampicillin recovered from lakes in Brazil and from wastewater treatment plants in China [15, 32, 33]. Also, *Aeromonas hydrophilia* isolated in Limpopo Province of South Africa was found to be positive for *bla*_*TEM*_ gene [34]; however, our study is the first report on the presence of *bla*_*TEM*_ gene in *Aeromonas* isolates from Eastern Cape Province of South Africa. In another study, three *Aeromonas hydrophila* strains isolated from patients blood in Taiwan were found to harbour *bla*_*TEM*_ gene [35], and another report of *bla*_*TEM*_ gene positive *Aeromonas hydrophilia* from an aged patient with necrotizing fasciitis [36] and fecal *Aeromonas caviae* from a patient with intestinal ischemia in France [35, 37], has been documented. The presence of *β*-lactamase gene in both clinical and environmental isolates of *Aeromonas* species is worrisome as it tends to limit treatment options in *Aeromonas* infections. This study further corroborates wastewater as reservoir of antibiotic resistance determinants in the environment. 

## 5. Conclusion

In this study, high incidence of multiple antibiotic resistance amongst *Aeromonas *species was observed suggesting wastewater as a reservoir of antibiotic resistance determinants in the study communities. The need to ensure that discharged final effluents of wastewater treatment plants are adequately treated to remove such pathogens as *Aeromonas* species is here advocated to prevent the dissemination of multidrug-resistant determinants into the receiving waterbodies.

## Figures and Tables

**Table 1:** Sequence of primers used for detection of antibiotics resistance genes.

Primers	Sequence (5^′^ to 3^′^)	Target gene	Reference
pse-F	ACC GTA TTG AGC CTG ATT TA	*blaP1* class A *β*-lactamase	[22]
pse-R	ATT GAA GCC TGT GTT TGA GC		

Class 1 intg F	GGC ATC CAA GCA GCA AG	Class 1 integron	[23]
Class 1 intg R	GGC ATC CAA GCA GCA AG		

Class 2 int F	CGG GAT CCC CGG CAT GCA CGA TTT GTA	Class 2 integron	[23]
Class 2 int R	GAT GCC ATC GCA AGT ACG AG		

*bl* *a*_*TEM*_ F	AGGAAGAGTATGATTCAACA	*bl* *a*_*TEM*_	[24]
*bl* *a*_*TEM*_ R	CTCGTCGTTTGGTATGGC		

*Tet*(*C*)-1	GGT TGA AGG CTC TCA AGG GC	*TetC*	[18]
*Tet*(*C*)-2	GGT TGA AGG CTC TCA AGG GC		

**Table 2:** Distribution of antibiotic-resistant genes in *Aeromonas* species.

Antibiotic-resistant gene	Distribution (%)
*bl* *aP*1class A *β*-lactamase	20.8%
*bl* *a*_*TEM*_	8.3%
*TetC*	0
Class 1 integron	20.8%
Class 2 integron	0
